# Ab Initio Study of the Structures, Bonding Interactions, and Thermal Stability of the Li-Decorated 2D Biphenylene Sheet

**DOI:** 10.3390/nano15090700

**Published:** 2025-05-07

**Authors:** María Begoña Torres, Alexandre Lebon, Luis Enrique González, Luis Javier Gallego, Andrés Vega

**Affiliations:** 1 Departamento de Matemáticas y Computación, Escuela Politécnica Superior, Universidad de Burgos, ES-09006 Burgos, Spain; 2 Laboratoire de Chimie Electrochimie Moléculaire et Chimie Analytique, Université de Brest, UMR CNRS 6521, F-29285 Brest, France; alexandre.lebon@univ-brest.fr; 3 Departamento de Física Teórica, Atómica y Óptica, Facultad de Ciencias, Universidad de Valladolid, ES-47011 Valladolid, Spain; luisenrique.gonzalez@uva.es (L.E.G.); avega@uva.es (A.V.); 4 Área de Física de la Materia Condensada, Departamento de Física de Partículas, Facultad de Física, Universidad de Santiago de Compostela, ES-15782 Santiago de Compostela, Spain

**Keywords:** 2D materials, biphenylene, Li-adsorption on biphenylene, structural properties, electronic properties, stability at room temperature, ab initio density-functional theory, ab initio molecular dynamics, quantum chemical topology, metallic bonding

## Abstract

We performed an extensive study on the most stable structures, the electronic properties, and the thermal stability of the 2D biphenylene sheet decorated with Li atoms. Our structural results show that the Li storage capacity of biphenylene is much higher than that recently reported, which increases the interest in this 2D material as a promising anode material for Li-ion batteries, although Li diffusion is not expected at room temperature. Moreover, we found striking phenomena that had not been detected yet, such as the formation of Li zigzag wires and metallic Li monolayers on the biphenylene sheet beyond a certain coverage threshold. In our calculations, we use high-level density-functional theory, quantum chemical topology analysis, and ab initio molecular dynamics simulations. In particular, the latter methodology allows for confirming the stability of the predicted Li-decorated biphenylene structures at room-temperature conditions.

## 1. Introduction

A wide variety of two-dimensional (2D) carbon allotropes have been theoretically predicted, and some have already been successfully synthesized (see, e.g., Refs. [1,2,3,4,5,6,7]). Among them, the biphenylene (BP) sheet has attracted particular attention. Following several theoretical predictions [8,9,10,11,12,13,14], Fan et al. recently synthesized nanoribbons of the BP sheet—a non-benzene 2D carbon allotrope composed of adjacent octagonal (o), hexagonal (h), and square (s) rings of sp^2^-hybridized carbon atoms—using an on-surface interpolymer dehydrofluorination (HF-zipping) reaction [15] (see also Ref. [16]). Since then, extensive studies have been conducted to explore its electronic, mechanical, and thermal properties [17,18,19,20,21,22,23,24,25,26,27,28,29]. Notably, Demirci et al. [29] demonstrated that not only the C-ohs structure is mechanically and thermally stable, but also ohs monolayers of other group IV elements, IV−IV, III−V, and II−VI compounds, along with their multilayers and corresponding 3D periodic ohs crystals. These materials represent a novel class with distinct physical and chemical properties compared to their hexagonal counterparts [29]. Three-dimensional porous carbon allotropes composed of biphenylene nanoribbons and single-wall carbon nanotubes, which act as linkers, have also been predicted to be stable and have properties of interest [30].

In general, pristine carbon-based nanostructures are weakly reactive. However, their reactivity can be significantly enhanced through functionalization with suitable species. For instance, computational studies have shown that lithium (Li) doping increases the reactivity of carbon nanotubes, graphene, and more complex carbon nanostructures [31,32,33]. Understanding the interaction of Li atoms with 2D carbon allotropes is, therefore, of fundamental interest and potential technological relevance. In this context, we recently investigated the Li decoration of popgraphene, a 2D system consisting of pentagonal-octagonal-pentagonal (5-8-5) carbon rings [34]. Although pographene has yet to be synthesized, the controlled fabrication of extended “5-5-8” line defects in graphene suggests its possible realization [35], and theoretical calculations have confirmed its remarkable dynamic, thermal, and mechanical stability [6].

In the present work, we perform a detailed investigation of the Li-decorated BP sheet using ab initio density functional theory (DFT) calculations. This general methodology, sometimes in conjunction with other approaches such as the phase field model [36], provides a powerful tool for describing a wide range of materials and low-dimensional systems of complex morphology and composition. Apart from its fundamental interest, our study is motivated by the potential application of BP sheets as efficient carbon-based anode materials for Li-ion batteries. Recently, Chen and Dhanthala [37] used DFT to analyze various Li concentrations on the BP substrate, focusing on the most stable adsorption sites and their impact on anode capacity. Here, we expand upon their work by exploring a broader range of possible Li adsorption sites, including mixed configurations such as hexagon–octagon and hexagon–square arrangements. We systematically increase the Li concentration up to the formation of what resembles a sort of adsorbed Li monolayer. All structures undergo full relaxation, including atomic positions and unit cell translation vectors.

Additionally, we perform a quantum topological analysis of key scalar fields such as charge density and the electron localization function to uncover the bonding patterns associated with different local environments created by Li adsorption. To further assess the structural stability under practical conditions, we perform ab initio molecular dynamics (MD) simulations for selected configurations, evaluating their dynamic behavior at finite temperatures. Our results suggest that the BP sheet can accommodate a high Li content with favorable adsorption energies and that the BP-based anode could achieve a significantly higher capacity than that previously predicted by Chen and Dhanthala [37], although Li diffusion is not expected at room temperature.

The remainder of this paper is structured as follows. Section 2 outlines the details of the methodology employed. Section 3 is devoted to the results. Structural arrangements for different Li concentrations, along with the key structural parameters and binding energies, are presented in Section 3.1. In Section 3.2, we discuss our results in the light of the quantum topological analysis. Section 3.3 is devoted to analyzing the dynamic stability. Finally, Section 4 summarizes the main conclusions of this work.

## 2. Materials and Methods

We performed the DFT calculations using the Vienna Ab Initio Simulation Package (VASP) [38,39], which employs a plane-wave basis set to expand the orbitals. A cutoff energy of 500 eV was chosen for the plane-wave basis set. For the exchange and correlation (XC) potential, we used the RevPBE+D3 approximation with Becke–Johnson damping within the D3 correction [40]. Van der Waals (VdW) corrections significantly improve the accuracy of GGA XC functionals in describing atom adsorption on graphene, leading to more precise adsorption energies and distances. Among the various PBE versions, RevPBE+D3 has been shown to be the most accurate in this regard [41]. Originally, RevPBE was demonstrated to systematically improve atomization energies over PBE for a broad database of small molecules [42]. Subsequent studies also showed that RevPBE enhances the chemisorption energetics of atoms and molecules on transition-metal substrates [43]. Additionally, recent research indicates that for alkali atom adsorption on graphene, the performance of VdW functionals is largely independent of the choice of the pseudopotentials when RevPBE is used [44], a desired quality for the results to be reproducible across different DFT codes. In the particular case of systems that include Li and C atoms, such as Li intercalated graphite compounds, previous studies have shown that revPBE+D3 with Becke–Johnson damping produces results for structural, energetic, electronic and defect properties in good agreement with experimental data [45].

The core interactions were treated using the projector-augmented wave (PAW) method [46]. In particular, we used the “sv” version of the PAW potentials distributed with VASP for Li, which includes the three electrons of Li as valence states and the standard PAW potential for C, with 4 valence electrons. A k-grid of 6 × 6 × 1 was used for Brillouin zone integrations after testing k-point convergence, and we ensured sufficient separation (30 Å) between periodic images in the normal direction to prevent spurious interactions. Structural relaxations were performed until the residual force on each atom was below 0.01 eV/Å and external pressure was minimized. The electronic convergence criterion for total energy calculations was set to 10^−4^ eV per unit cell.

Once structural information was obtained with VASP, further analysis was conducted to understand how the system components interact. Quantum Chemical Topology (QCT) analysis provides an elegant approach to this task. Within QCT, various scalar fields can be analyzed based on their gradient properties. Here, we focused on two key scalar fields: the electronic density and the electron localization function (ELF). By analyzing the electronic density, the nature of bonding can be inferred from the properties of critical points. In this study, we considered bond critical points (BCPs) and did not examine other types, such as ring or cage critical points [47]. BCPs are saddle points in the electronic density, characterized by a single density minimum along the bond path and two maxima in perpendicular directions. These features allow for distinguishing different types of bonding, including covalent, ionic, polar, dative, metallic, VdW, and hydrogen bonds [47,48]. The bond classification relies on key parameters at the BCPs, including electronic density, Laplacian of the density, and energy. Their atomic units (a.u.) are, respectively, e/Bohr^−3^, e/Bohr^−5^, and Ha/Bohr^−3^. Matta [48] classifies bonds into shared (covalent, polar-covalent) and closed-shell (dative, ionic, metallic, VdW) categories. In this work, we focus on BCP values for interactions between Li and C atoms in the 2D monolayer, as well as Li-Li interactions.

To contextualize these values, we briefly outline typical BCP properties for different bond types. Covalent bonds usually exhibit an electronic density above 0.2 a.u. at the BCP, with a negative Laplacian between −0.5 and −1.0 a.u., indicating electron density accumulation. Ionic bonds, in contrast, have lower electronic densities at the BCP (typically one order of magnitude smaller than covalent bonds) and a positive Laplacian (0.1–0.2 a.u.), signifying charge depletion. The energy at the BCP is generally negative for covalent bonds and positive for ionic bonds.

For further analysis, we prepared 2 × 2 replicas of the optimized VASP cells and performed all-electron single-point energy calculations using the Gaussian16 quantum chemistry package [49], employing the same DFT functional with a 3–21G* basis set. The rationale for not using PAW potentials to extract quantum topological information is discussed in detail in previous work by some of the authors [34]. Unlike that work, we include ELF analysis here. ELF was originally introduced in the early 1990s [50] as a measure of the likelihood of finding two electrons with opposite spins in a given molecular region. Its importance in quantum topology was later emphasized by Silvi and Savin [51], who applied a gradient analysis to this scalar field. They demonstrated that ELF could classify chemical bonds. Subsequently, they proposed a method for quantifying electron populations within selected ELF-defined regions, known as basins. These basins, which are associated with nuclei, can be categorized as core basins (restricted to a single nucleus) or valence basins (shared between nuclei). In covalent bonds, the valence basin is shared between two atoms, and bond identification is achieved by analyzing the electron population and its variance [52]. In contrast, ionic bonds exhibit no basin between the atoms and lack an attraction center. Hence, electronic density and BCP analysis provide complementary insights into bond characterization.

The general methodology is as follows. Gaussian16 calculations generate wavefunctions, which are then mapped onto a spatial grid for electronic density and ELF analysis. Post-processing of electronic density data is conducted using the DGRID5.1 code [53], extracting BCPs and their characteristics. Similarly, ELF gradient analysis is performed using the TOPMOD tool [54].

A final check on the stability of the considered structures is carried out using the VASP package to perform MD simulations at a temperature of 300 K. The initial ionic velocities are sampled from a Maxwell–Boltzmann distribution, and subsequently the temperature is maintained by rescaling the ionic velocities at each MD step to yield an ionic kinetic energy that corresponds to the desired temperature. The simulations, for each structure considered, cover a total of 3000 time steps of 1 fs. During this time, the ions may vibrate around their equilibrium positions, indicating a dynamically stable structure, or migrate into new positions to form a different structure after passing, thanks to the thermal energy, over possible barriers that separate the original and final structures. In fact, some of the structures considered below were obtained after the relaxation of final configurations in MD runs, which yielded lower energies than the initial one.

## 3. Results

### 3.1. Structural Configurations of Li Atoms Adsorbed on Biphenylene

**Figure 1:**
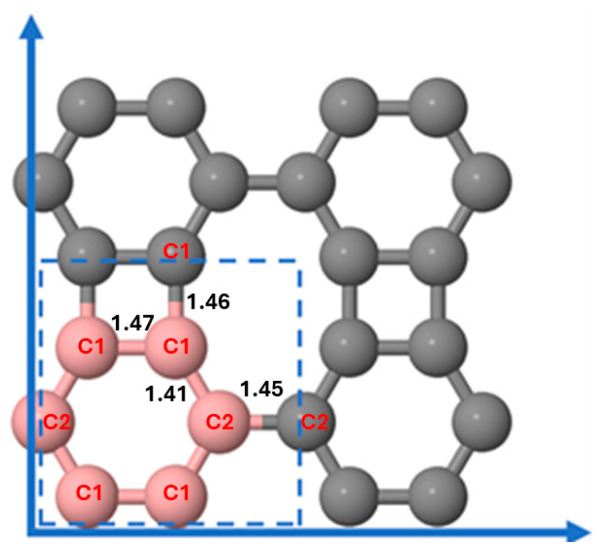
The primitive cell of the BP sheet (bounded by a rectangle with dashed lines, with atoms in pink) and structure of the 2 × 2 × 1 supercell used in our calculations after relaxation. Lattice vectors are indicated in blue color. The numbers are atom–atom distances in Å.

We discuss the predicted structures of the pristine and the Li-decorated BP sheets. Pristine BP is a monolayer with the crystallographic point group D2h, with all carbon atoms lying in the same plane. The sheet contains three types of rings: hexagonal, octagonal, and square. The primitive cell of the BP sheet (shown in Figure 1, bounded by a rectangle with dashed lines) has six C atoms, with lattice constants a = 4.53 Å and b = 3.78 Å, consistent with the results reported by Liu et al. [55]. 

Figure 1 displays the 2 × 2 × 1 supercell structure containing 24 carbon atoms used in our calculations, with interatomic distances obtained after relaxation. As shown in Figure 1, the computed C-C bond lengths are 1.47 and 1.46 Å (C1-C1 bonds), 1.41 Å (C1-C2 bond), and 1.45 Å (C2-C2 bond), which agree well with previous computational results [55].

As mentioned above, one of the main objectives of this study is to find the stable and optimal decorations of the BP sheet with Li atoms. The average binding energy of Li atoms on the sheet is calculated using the equation

(1) $$E_{b}={(E}_{BP}+{nE}_{Li}-E_{nLi-BP})\;/n\;$$

where $E_{BP}$, ${E_{Li},\;and\;E}_{nLi-BP}$ are the energies of pristine BP, that of an isolated Li atom, and that of the optimized BP structure upon adsorption of *n* Li atoms, respectively. A more positive value of $E_{b}$ indicates a stronger interaction between the Li atoms and the BP sheet.

We examined the Li adsorption on the BP sheet by progressively adding Li atoms. Since both sides of the BP sheet can interact with Li atoms, we investigated adsorption on a single side and on both sides of the sheet. Various adsorption sites were explored, including hexagonal, octagonal, and square hole sites, as well as combinations of them. Additionally, we studied configurations where the Li atoms were uniformly distributed across the BP sheet and cases where, particularly at lower Li concentrations, Li atoms clustered together, leading to an inhomogeneous distribution. While increasing the number of Li atoms on the BP sheet, we focused only on even numbers of Li atoms and took advantage of symmetry conditions. For each case, multiple initial structural candidates were considered for optimization.

**Figure 2:**
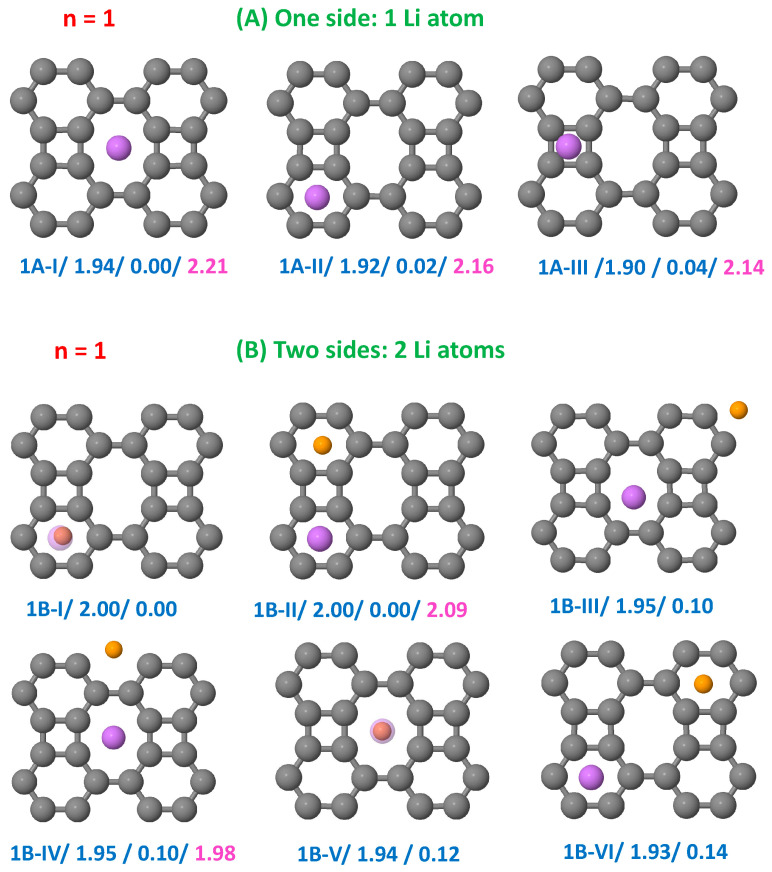
Configurations of the hybrid BP/Li system with a Li atom on a single side of the sheet panels (**A**) and with a Li atom on each side of the sheet panels (**B**). Each configuration is labeled as 1X-Y, with X = A or B, and Y = I, II, III, etc., reflecting the order of stability of the structures. The first number below each configuration corresponds to the binding energy (in eV), and the second number corresponds to the total energy of the configuration (in eV) relative to that of the most stable structure. In some cases, we also show a third number, which is the binding energy reported in Ref. [37] for such structures. The Li atoms on the front side of the BP sheet have a greater size (and different color) than the Li atoms on the rear side.

**Figure 3:**
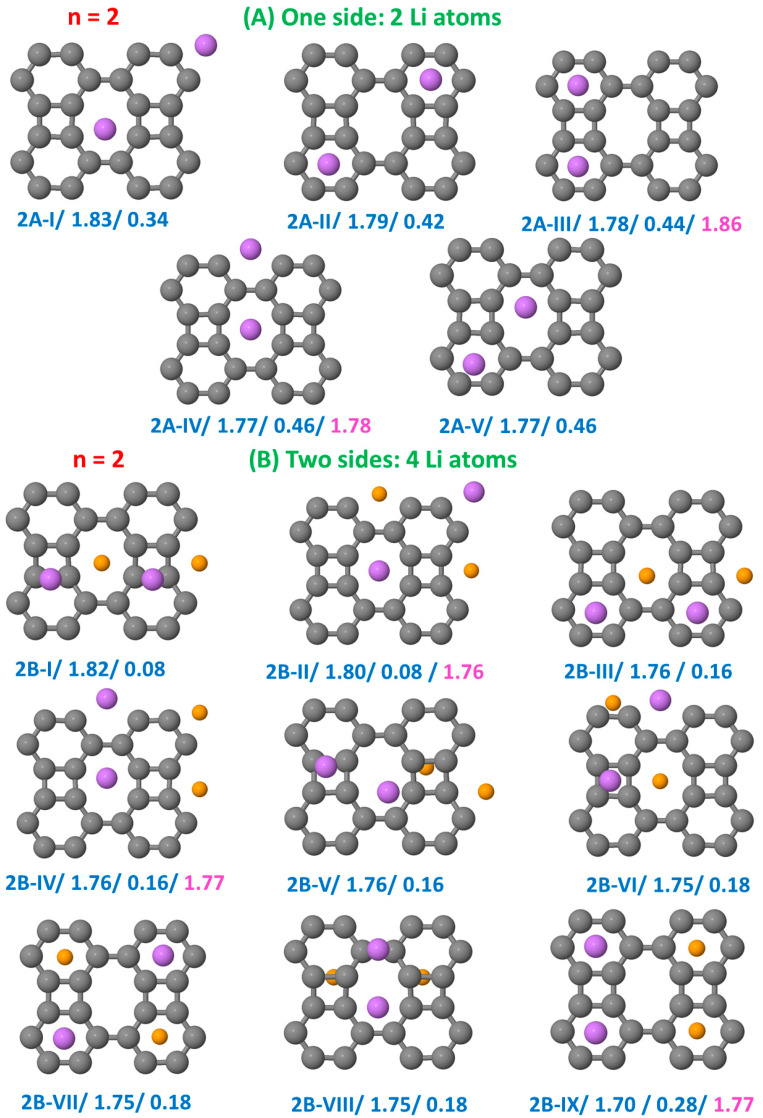
Configurations of the hybrid BP/Li system with two Li atoms on a single side of the sheet panels (**A**) and with two Li atoms on each side of the sheet panels (**B**). Each configuration is labeled as 2X-Y, with X = A or B and Y = I, II, III, etc., reflecting the order of stability of the structures. The first number below each configuration corresponds to the binding energy (in eV), and the second number corresponds to the total energy of the configuration (in eV) relative to that of the most stable structure. In some cases, we also show a third number, which is the binding energy reported in Ref. [37] for such structures. The Li atoms on the front side are of a greater size (and different color) than the Li atoms on the rear side.

**Figure 4:**
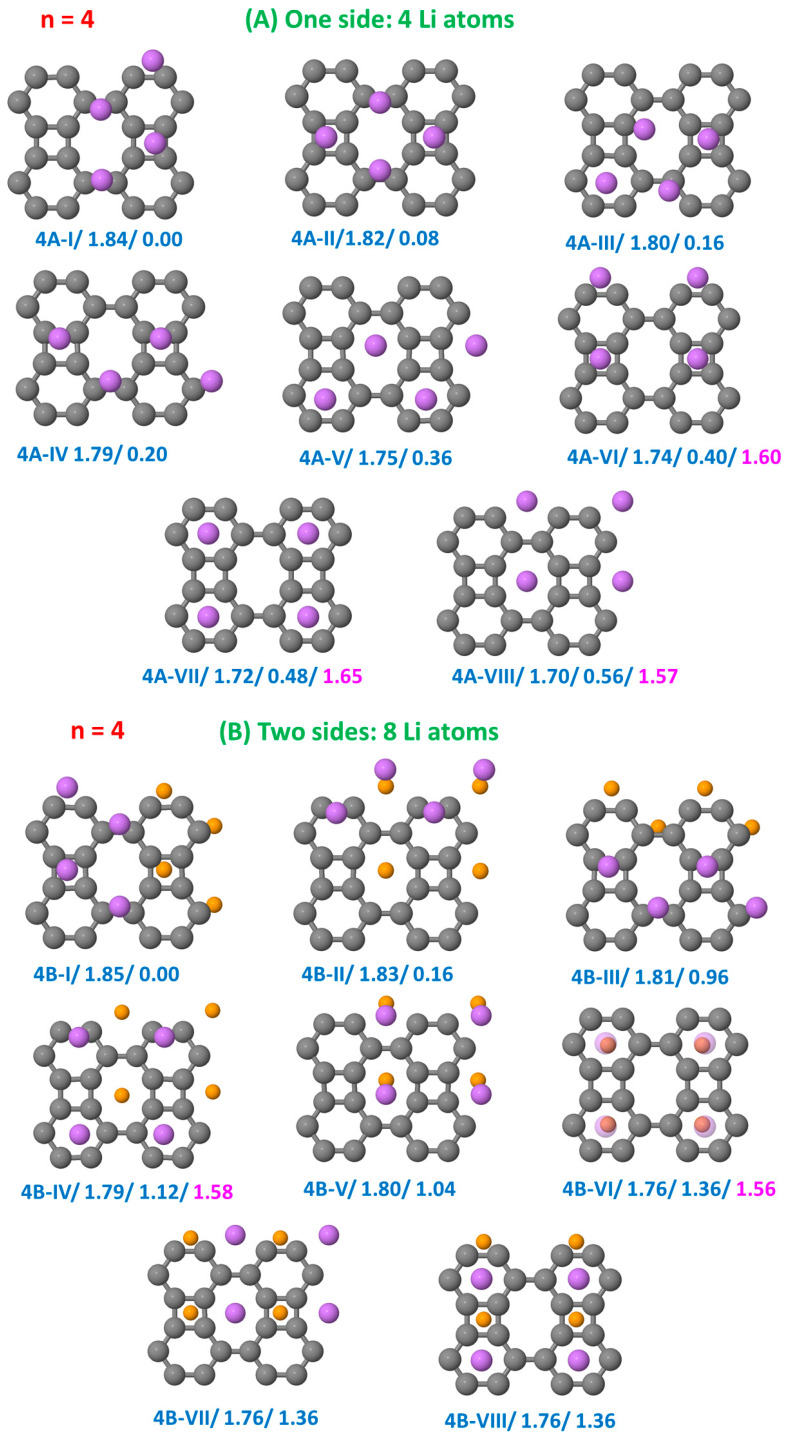
Configurations of the hybrid BP/Li system with four Li atoms on a single side of the sheet panels (**A**) and with four Li atoms on each side of the sheet panels (**B**). Each configuration is labeled as 4X-Y, with X = A or B and Y = I, II, III, etc., reflecting the order of stability of the structures. The first number below each configuration corresponds to the binding energy (in eV), and the second number corresponds to the total energy of the configuration (in eV) relative to that of the most stable structure. In some cases, we also show a third number, which is the binding energy reported in Ref. [37] for such structures. The Li atoms on the front side are of a greater size (and different color) than the Li atoms on the rear side.

**Figure 5:**
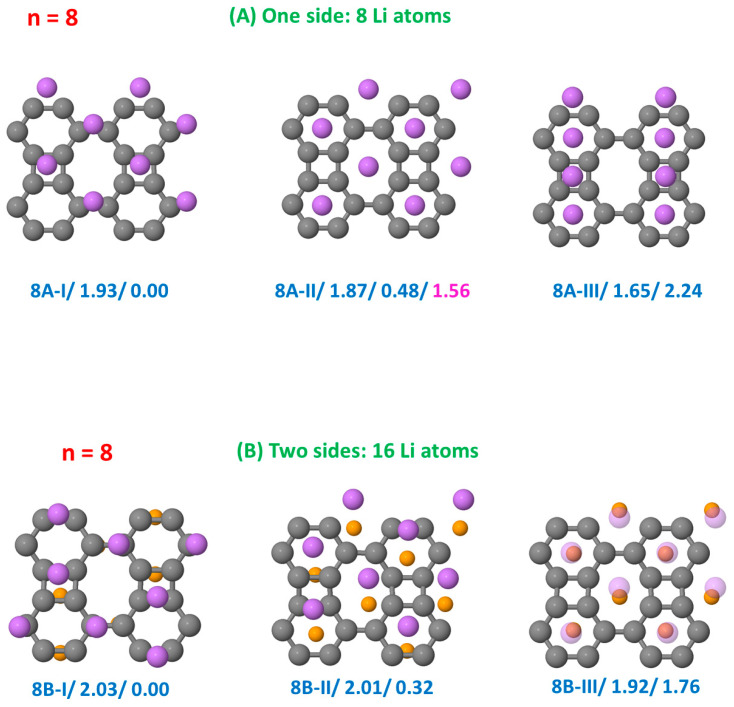
Configurations of the hybrid BP/Li system with eight Li atoms on a single side of the sheet panels (**A**) and with eight Li atoms on each side of the sheet panels (**B**). Each configuration is labeled as 8X-Y, with X = A or B and Y = I, II, III, etc., reflecting the order of stability of the structures. The first number below each configuration corresponds to the binding energy (in eV), and the second number corresponds to the total energy of the configuration (in eV) relative to that of the most stable structure. In the same case, we also show a third number, which is the binding energy reported in Ref. [37] for such a structure. The Li atoms on the front side are of a greater size (and different color) than the Li atoms on the rear side.

Figure 2, Figure 3, Figure 4 and Figure 5 show the results obtained for the adsorption of *n* = 1, 2, 4, and 8 Li atoms on a single side (top panels) and on both sides (bottom panels) of the BP sheet. Each configuration is labeled as nX-Y, where n represents the number of Li atoms on a single side, X = A or B indicates whether the Li atoms are adsorbed on a single side or on both sides of the sheet, respectively, and Y = I, II, III, etc., denote the most stable configurations obtained for each nX case, ordered by increasing order of stability. In Figure 2, Figure 3, Figure 4 and Figure 5, we include two values below each configuration. The first corresponds to the binding energy (in eV), allowing comparison between configurations with different Li concentrations, and the second represents the total energy of the configuration (in eV) with respect to that of the most stable structure. For comparison, a third value is added in the cases in which binding energies have previously been reported [37].

A single Li atom binds on octagonal, hexagonal, and square hole sites of the BP supercell, as shown in Figure 2A, with binding energies of 1.94 eV, 1.92 eV, and 1.91 eV, respectively. These values exceed the cohesive energy of bulk Li (1.63 eV [56]), indicating strong adsorption. For comparison, the binding energy of a Li atom on graphene is 1.096 eV [57]. The equilibrium distances between the Li atom and the BP sheet are 1.70 Å (hexagon), 1.60 Å (octagon), and 1.80 Å (square).

Figure 2B illustrates the optimal configurations when placing one Li atom on each side of the sheet. The first row shows cases in which the Li atom occupies hexagonal hole sites, while the second row shows configurations with the Li atom occupying octagonal hole sites. In the ground state, the Li atoms are positioned in opposing hexagons. This arrangement is nearly degenerated with the structure 1B-II, which was proposed by Chen and Dhanthala [37]. In structure 1B-II, the Li atoms occupy adjacent hexagons on opposite faces of the sheet. When the Li atoms are placed in the most distant hexagons (structure 18-VI), the binding energy decreases by 0.072 eV per atom. The remaining structures—1B-III, 1B-IV, and 1B-V—correspond to cases in which the Li atoms are located in octagonal hole sites. 

Figure 3 presents the different configurations for n=2. When the Li atoms are adsorbed on a single side of the sheet, they preferentially occupy octagonal hole sites, with configuration 2A-I being the most stable. The configuration in which the Li atoms occupy hexagonal hole sites (2A-II) has an energy very close to that of 2A-I. In general, the structures in which the Li atoms are more separated (2A-I and 2A-II) are energetically favored over those in which they are close together (2A-III and 2A-IV), as proximity leads to Li-free regions within the lattice. Our results indicate a clear tendency to avoid Li clustering for n = 2. Additionally, we identify a novel configuration in which the Li atoms are adsorbed, forming a hexagon–octagon cluster (2A-V). In this structure, the Li atoms are separated by a distance d = 3.41 Å, with the Li atom in the octagonal position slightly displaced from the center of the ring. This configuration emerges from an initial structure in which the Li atoms were placed closer together, with an initial separation d(Li-Li) = 2.9 Å.

When two Li atoms are adsorbed on each side of the sheet, the most stable configuration (2B-I) corresponds to an arrangement in which the Li atoms on opposite sides of the layer tend to align. In the second (2B-II) and fourth (2B-IV) most stable configurations, the Li atoms occupy well-separated and adjacent octagonal hole sites, respectively. Configuration 2B-III exhibits a zigzag arrangement of Li atoms. A similarity in energy between structures 1A-I and 1A-II suggests a consistent pattern in Li adsorption. This type of configuration, in which the Li atoms occupy both hexagonal and octagonal hole sites, has not been previously explored. Four additional isomers (2B-V to 2B-VIII) exhibit energies close to that of 2B-IV. In configurations 2B-V and 2B-VI, the Li atoms occupy octagonal and square hole sites. A variety of additional structures, not shown here, fall within the same energy range and correspond to different Li arrangements in those hole sites, depending on whether they are positioned above or below the sheet. Furthermore, the Li atoms show a preference for occupying well-separated hexagonal hole sites (2B-VII) rather than adjacent ones (2B-IX), which is consistent with the behavior observed for single-sided adsorption. Configuration 2B-VIII shows nearby Li atoms. 

Figure 4 presents the optimized configurations for *n* = 4 Li atoms. When adsorbed on a single side of the sheet, the most stable configuration (4A-I) exhibits extended Li wires arranged in a zigzag pattern, with the Li atoms occupying bridge positions over octagonal and square hole sites. A similar wire-like structure is also observed in the isomer 4A-IV but rotated by 90° relative to the 4A-I configuration. To our knowledge, these Li wire formations on a BP layer have not been reported previously. Configurations 4A-II and 4A-III reveal the formation of compact Li_4_ clusters on the BP sheet, contrasting with the more extended wire-like arrangements. In the remaining isomers, the Li atoms are more evenly distributed across the surface. Notably, configuration 4A-V consists of Li atoms adsorbed on both hexagonal and octagonal hole sites, evolving from the previously identified 2A-V and 2B-III structures. In this arrangement, the Li atoms form zigzag chains, resulting in an uneven distribution where certain lattice rows contain Li while others remain vacant. The Li–Li separation in this configuration is d(Li-Li) = 3.32 Å. Other configurations involve Li adsorption in specific hole sites: Li atoms occupy exclusively square (4A-VI), hexagonal (4A-VII), or octagonal (4A-VIII) hole sites, with Li–Li distances of 4.53 Å and 3.77 Å in all three cases.

For adsorption on both sides of the BP sheet (Figure 4, panels B), the two most stable configurations, 4B-I and 4B-II, exhibit Li wire formations like those in 4A-I and 4A-III, respectively, but extending across both sides of the structure. In the next most stable configuration (4B-III), the Li atoms are placed on hexagonal and octagonal hole sites, resulting in a more uniform Li distribution due to the higher Li surface concentration. A nearly degenerate configuration, 4B-IV, consists of Li atoms occupying opposing octagonal hole sites, followed closely in stability by 4B-V, where the Li atoms are positioned in facing hexagons. Less stable configurations include 4B-VI and 4B-VII, where the Li atoms are adsorbed in square–hexagon and square–octagon combinations, respectively, both exhibiting comparable binding energies.

For *n* = 8, we examined three configurations with the Li atoms adsorbed on a single side of the sheet (Figure 5, panels A): (i) on hexagonal–octagonal hole sites, (ii) on hexagonal–square hole sites, and (iii) on octagonal–square hole sites. From the latter, we derived structure 8A-I, where the Li atoms initially positioned on octagons have shifted toward the bridge sites of these octagons, while the remaining Li atoms remain on square sites. This configuration exhibits a high binding energy of 1.93 eV, forming a sort of monolayer of Li atoms with an interatomic distance of d(Li-Li) = 3.0 Å. Close in energy, at 1.87 eV, is the structure 8A-II, in which the Li atoms occupy hexagonal–square hole sites, evolving from the growth of 2A-V and 4A-V. In contrast, the structure 8A-III shows a significantly lower binding energy, indicating a less favorable adsorption configuration. 

Panels B of Figure 5 illustrate the adsorption of sixteen Li atoms on the BP sheet, with eight atoms on each side. We analyzed three configurations: (i) a Li monolayer derived from the 8A-I configuration but extending to both sides of the sheet; (ii) two Li monolayers, one on each side of the sheet, shifted in opposite directions to those of the previous structure, 8A-I; and (iii) a distribution of the Li atoms occupying hexagonal and octagonal hole sites, corresponding to the configuration 8A-II on both sides. The first configuration is the most stable. We find that in the three structures, the Li layer below the BP sheet is equivalent to a 180-degree rotation of the layer above about an axis in the x direction passing through the cell center. 

We note that the ground states proposed by Chen and Dhanthala [37] for most BP/Li systems differ significantly from those obtained in our calculations. Their proposed ground-state configurations correspond to our second (1B-II, nearly degenerated), third (2A-III), fourth (2B-IV), seventh (4A-VII), fourth (4B-IV), and second (8A-II) most stable structures for increasing Li coverage. In all cases, the most stable configuration identified in our study was not considered in their analysis. The broader range of input configurations explored in our work enabled us to identify lower-energy structures and propose more plausible ground states.

To further investigate possible clustering effects, we also examined the stability of the BP/Li system by considering an adsorbed Li_2_ dimer. The Li_2_ dimer (d(Li-Li) = 2.7 Å) was placed parallel to the BP sheet on octagonal–hexagonal (I) and octagonal–square (II) hole sites. After relaxation, the Li atoms became separated, with interatomic distances d(Li-Li) = 3.15 Å and d(Li-Li) = 2.93 Å, respectively. The binding energies of these structures are 1.74 eV and 1.75 eV, which are lower than those obtained for structures 2A-I to 2A-V (1.83–1.77 eV, shown in Figure 3). This suggests that the Li atoms tend to remain dispersed rather than forming stable dimers. Additionally, if a free Li_2_ dimer is initially placed above the BP sheet and is allowed to relax, it dissociates spontaneously upon approaching the surface without encountering any energy barrier. This occurs regardless of the initial orientation of the Li_2_ dimer. When the dimer is placed perpendicular to the sheet on hexagonal or octagonal hole sites, the Li atoms separate to distances of 3.34 Å and 3.15 Å, respectively. The results of these calculations for two Li atoms suggest that Li atoms tend to disperse rather than form clusters on the BP sheet. However, as the Li concentration increases beyond two atoms, we observe a tendency for the Li atoms to organize into structures resembling nanowires and even monolayers on the substrate instead of more dispersed arrangements. In the following sections, we explore this phenomenon in detail, as it has not been previously reported and has implications for Li atom mobility.

Now, let us analyze in detail the variation in the average Li binding energy as a function of Li content. Our results show that, for low Li concentrations, these energies are comparable to those reported by Chen and Dhanthala [37] and remain well above the cohesive energy of bulk Li (1.63 eV [56]), which is the threshold used by these authors to consider favorable adsorption of Li on BP and to determine its capacity as an anode material. However, as the Li content increases, our calculated binding energies decrease at a slower rate than those obtained by these authors. Notably, even with 8 Li atoms adsorbed per unit cell on one side of the BP monolayer, our binding energies remain above the cohesive energy of bulk Li. In contrast, Chen and Dhanthala report binding energies falling below this threshold once more than 4 Li atoms are adsorbed. This suggests that if we take the cohesive energy of bulk Li as the minimum threshold for favorable Li adsorption, then our results indicate a significantly higher Li storage capacity for a BP-based anode than that previously predicted by Chen and Dhanthala [37]. It is important to note that this binding energy trend is not due to variations in exchange-correlation functionals, as we also tested the PBE functional and obtained the same trend. Another methodological difference is the choice of basis sets. We used a plane-wave basis set, which is effectively complete for the selected energy cutoff and free from the basis set superposition errors. The basis set used by Chen and Dhanthala [37] is not compatible with our code, preventing a direct cross-check. In the following section, we explore the nature of the bonding in these systems. As will be seen, with increasing Li content, our model captures the emergence of Li-Li interactions, which likely contribute to the overall stability of the system.

### 3.2. Quantum Chemical Topology Analysis

We now focus on understanding how the Li atoms organize on the BP monolayer to achieve stability. To this end, we have investigated the chemical bonding as Li atoms are added to the BP sheet. Here, one-sided structures serve as representative examples of the filling process of the BP layer with Li atoms. The application of Quantum Chemical Topology (QCT) tools provides insights into the nature of the chemical bonding between Li atoms and the BP sheet and sheds light on the superior stability observed for the structure decorated with eight Li atoms as compared to those decorated with four Li atoms.

**Figure 6:**
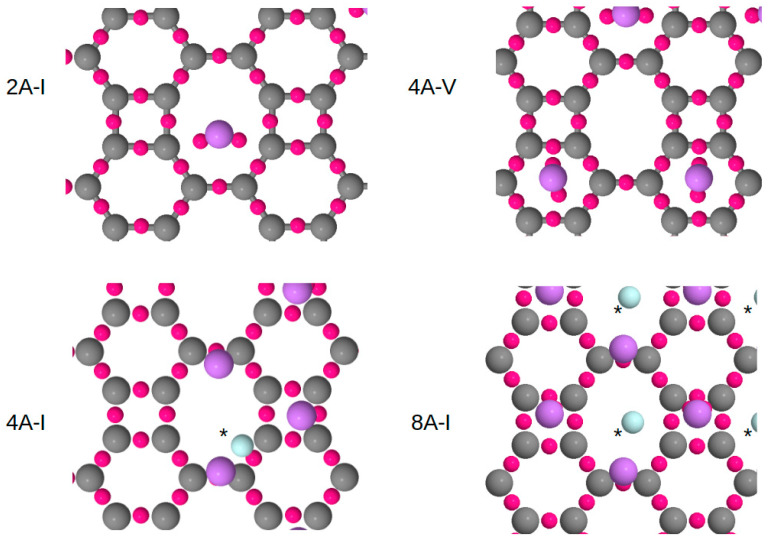
Bond critical points distribution for structures 2A-I, 4A-V, 4A-I, and 8A-I of the Li decorated BP sheet. Gray and violet balls are C and Li atoms, respectively. Small red balls are the BCPs. Asterisks denote that the adjacent light blue balls are metallic BCPs.

Figure 6 illustrates the bond critical points (BCPs), where C atoms are represented as grey balls, Li atoms as purple balls, and the small red balls correspond to the BCPs, whereas light blue balls stand for BCPs associated with the Li-Li interaction. These BCPs are first-order saddle points derived from the gradient of the electronic density, exhibiting a minimum along the bond direction—such as between two C atoms in a square motif—and two maxima along the perpendicular directions. Our analysis specifically focuses on the BCPs connecting Li atoms either to the BP layer or to each other. The complete set of BCP values is presented in Table 1, distinguishing between the Li-C ionic bond and the Li-Li metallic bond.

Ionic bonding between Li and C atoms is observed in all structures, with an electronic density value of approximately 0.02 a.u. (i.e., 20 × 10⁻^3^ a.u. in Table 1). These small values are characteristic of ionic interactions between Li and C atoms within the BP layer. The positive value of the Laplacian and the positive contribution to the energy at these points are additional evidence of the ionic nature of the Li-C bonding, which is in agreement with previous findings [34].

For the most densely decorated structure, 8A-I, the interaction between the Li atoms exhibits characteristics of metallic bonding, as indicated in Table 1. The presence of BCPs within the Li plane, with an electronic density value of 0.007 a.u. and a stabilizing energy contribution, together with an almost null Laplacian, confirms this metallic interaction. The relatively weak Li-Li bonding is also reflected in the interatomic separation of nearly 3.0 Å, a distance very close to the Li-Li separation in the body-centered cubic (bcc) structure of bulk lithium (3.04 Å, corresponding to a cubic cell parameter of 3.51 Å). Moreover, the 8A-I configuration exhibits an excellent match between the Li layer and the BP sheet, with only 1% epitaxial strain. The height of these BCPs relative to the BP layer is also reported in Table 1. The 4A-I structure featuring a zigzag wire of Li atoms exhibits a metallic bonding as well, with values of the density, Laplacian of the density, and energy quite similar to those observed for the 8A-I structure.

The most striking feature is the progressive onset of a metallic coverage that progressively installs as the Li atoms decorate the 2D BP layer. The growth of this metallic layer occurs in two steps. There is first evidence of a 1D ordering as the Li atoms are arranged in a zigzag way on top of the BP surface. The second step is the full coverage of the layer with the Li atoms arranged in a 2D layer; here, a continuous metallic layer extends across the BP surface. Remarkably, there is a common feature in the first and the second steps. The sites are, in fact, identical since one Li atom sits on top of a square hole site, whereas its first neighbor sits on the bridge of an octagonal hole site. Such metallic bonding was previously reported for Li decoration on popgraphene by some of us [34]. This indicates a common trend in 2D carbon allotropes: beyond a certain coverage threshold, the formation of a metallic surface acts as a stabilizing mechanism for the Li layer on the 2D carbon substrate.

We observe a clear evolution in the height and intensity of the BCPs as the BP surface becomes progressively filled with Li atoms. The height of the BCPs increases while their intensity—i.e., the electronic density value at the BCPs—decreases. This indicates a gradual weakening of ionic bonding, with the enhanced stability of the 8A-I structure primarily attributed to the emergence of Li-Li metallic bonding, which effectively replaces some of the ionic interactions. Evidence of this transition is provided by analyzing the number of ionic bonds per Li atom in the 2A-I and 4A-V structures, where each Li atom forms approximately two ionic bonds. In the 8A-I structure and the quite similar 4A-I structure, this number decreases to 1.2 ionic bonds per Li atom, while metallic Li-Li bonds appear with an average of 0.5 bonds per Li atom.

**Figure 7:**
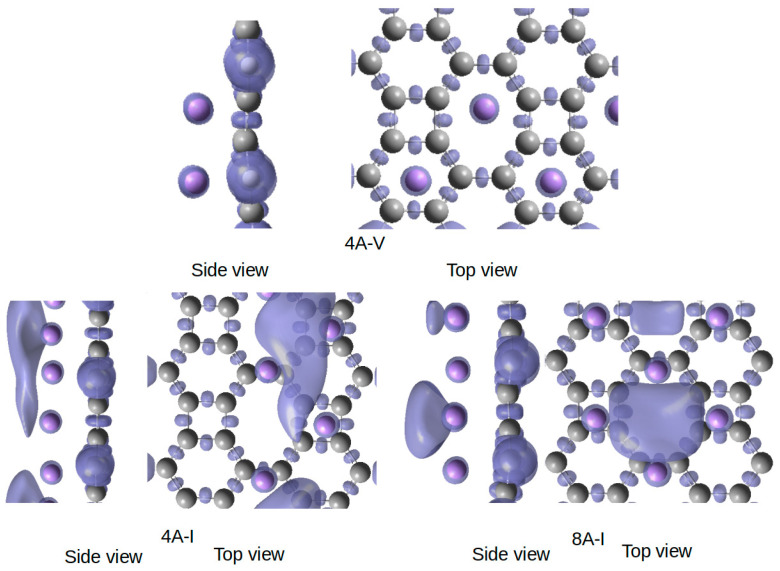
ELF plot of 4A-V, 4A-I, and 8A-1 structures. Gray and purple balls are for C and Li atoms, respectively. The ELF isosurface at ELF = 0.8 is shown in a transparent blue.

The emergence of metallicity in the 8A-I structure, where the Li atoms are located on the (octagonal) bridge and square hole sites, can be further illustrated by analyzing the electron localization function (ELF) for structures 4A-V, 4A-I, and 8A-I. This is precisely the purpose of Figure 7, which presents the ELF at an isosurface value of 0.8. The ELF is a scalar field ranging from 0 to 1, where higher values indicate regions of strong electron localization. The ELF plots for structures 4A-I and 8A-I reveal the presence of basins above the Li atoms —an indication of delocalized electronic states—whereas such basins are absent in structures 4A-V. For conciseness, a similar ELF plot for structures 2A-I is not shown, but it exhibits the same absence of basins as 4A-V. Structure 8A-I thus presents a characteristic ELF pattern typical of a carbon allotrope decorated with a Li layer. A side view of structure 4A-V confirms the absence of ELF basins between the Li and underlying C atoms, which is expected in the case of ionic bonding. This is consistent with the previous analysis of electronic density at the BCPs. Similarly, no ELF basins appear in the interstitial region between the C framework and the Li atoms both in the 4A-I and 8A-I structures. This finding is further support to the picture of the ionic nature of Li-C interactions. However, a key difference is observed: additional ELF basins emerge between Li atoms within the decorated Li layer, signaling metallic bonding.

Using a delicate post-processing tool [54], we were capable of determining the number of electrons both in the zigzag wire (4A-I) and in the 2D layer (8A-I). It amounts to 3.4 and 4.5 electrons, respectively. These electrons are shared within the ELF basins, confirming the metallic character of the Li layer. This result aligns with the BCP analysis in Table 1 and Figure 6, particularly with the highlighted BCPs, which indicate Li-Li metallic interactions.

In conclusion, the QCT analysis highlights the particular stability of the 8A-I structure, where the Li layer is epitaxially aligned with the (110) plane of bulk lithium’s body-centered cubic (bcc) structure. This suggests that BP monolayers provide an ideal substrate for Li adsorption, facilitating the formation of a 2D metallic Li layer. 

### 3.3. Dynamical Stability of the Structures 

To elucidate the stability of the structures at room temperature, we carried out ab initio MD simulations for several representative examples of the BP/Li configurations.

#### 3.3.1. Li on One Side

MD calculations have been carried out for a small sample of structures with *n* = 1, 4, and 8 Li atoms on one side of the BP sheet. 

We performed MD calculations starting from the configurations with a single Li atom on the BP layer located on a hexagonal hole site (1A-I), an octagonal hole site (1A-II), and a square hole site (1A-III). All of them are stable at 300 K, showing that the thermal energy is not enough to overcome the barrier between any two configurations (which was found to be between 0.10 eV and 0.30 eV using the nudged elastic band method [58]) and, therefore, the Li-ion vibrates near its equilibrium position. The mobility within the octagon is larger than within the hexagon and square due to the larger space available.

**Figure 8:**
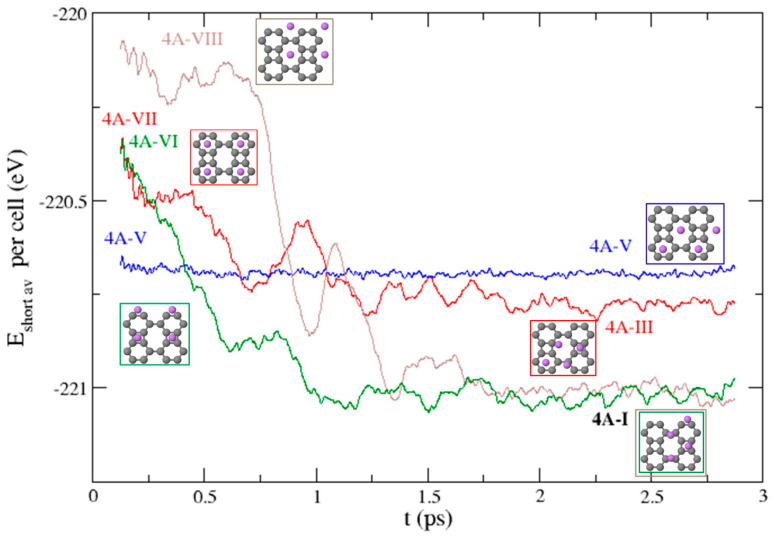
Time evolution of the short-time averages of the potential energy during the MD runs that start from the 4 higher energy structures that correspond to 4 Li atoms on one side of the BP sheet.

The structures obtained for *n* = 4 on a single side of the BP sheet have been allowed to evolve in MD runs, and the results show that the five structures from 4A-I to 4A-V remain stable during the 3 ps. On the contrary, structure 4A-VII converts to 4A-III in around 1.2 ps, whereas structures 4A-VI and 4A-VIII go into 4A-I in a similar time interval. This can be appreciated in Figure 8, which displays the time evolution of the short-time averages (0.25 ps long) of the potential energies of the systems starting at the V, VI, VII, and VIII configurations. It is interesting to note that the structures considered by Chen and Dhanthala [37] (VI, VII, and VIII) are not only high energy states but also dynamically unstable at room temperature.

**Figure 9:**
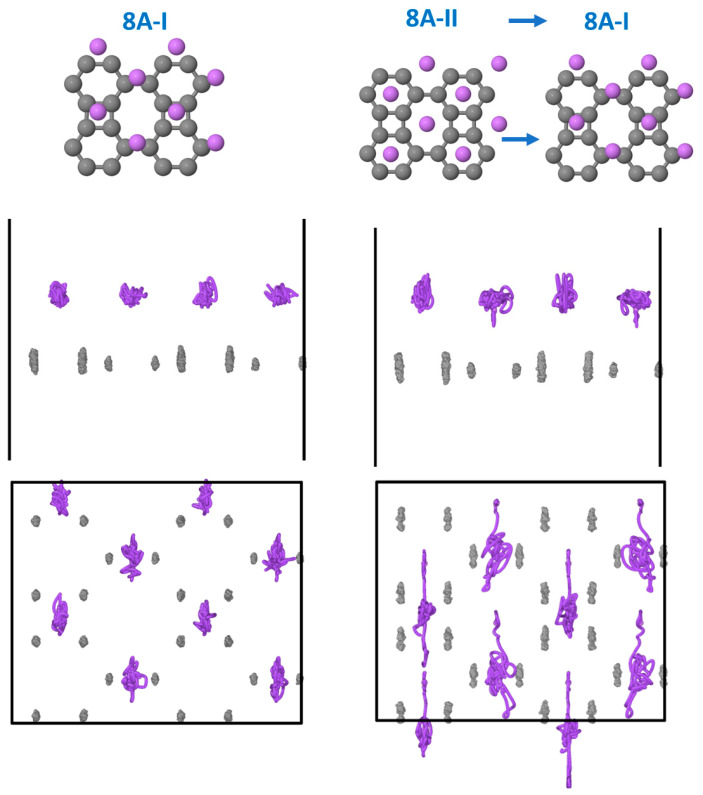
The top of the figure shows the initial and final configurations in the MD runs at 300 K, whose atomic trajectories are displayed directly below. Upper graphs: projections onto the xz plane; lower graphs: projections onto the xy plane. Left side graphs: MD starting from 8A-I; right side graphs: MD starting from 8A-II. Note that periodic boundary conditions are not applied to the trajectories to better observe the atomic motion.

For *n* = 8, the 8A-I configuration is found to be stable during the MD run, with half of the Li atoms remaining on the bridges of octagons and the other half on the square hole sites. However, the 8A-II configuration with Li on hexagons and octagons evolves to 8A-I. A video illustrating this evolution (movie-8A.mp4) can be found in the Supplementary Information. The MD run also allows for analyzing the atomic motion by following the trajectories of the atoms (see Figure 9). We observe that starting from configuration 8A-I, the C atoms only vibrate in the z-direction, while the amplitude of the vibration of the Li atoms in the y-direction is larger than in the x or z directions. However, starting from 8A-II, we observe additional vibration of the C atoms in the y-direction induced by the large displacements of the Li atoms. Note that the global transformation looks like a rigid shift of the metallic Li monolayer, discussed previously, in the y-direction, with the Li-Li distances changing on average less than 0.01 Å.

#### 3.3.2. Li on Both Sides

MD calculations have been performed for a small sample of structures with *n* = 2, 4, and 8 Li atoms on both sides of the BP sheet.

**Figure 10:**
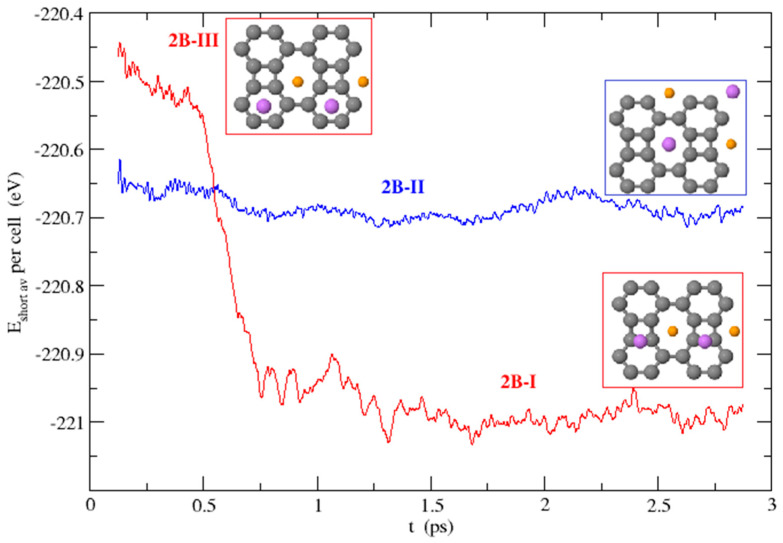
Time evolution of the short-time averages of the potential energy during the MD runs that start from the stable structure 2B-II (blue line) and from the dynamically unstable structure 2B-III (red line) that evolves to 2B-I.

In the first case, we started from the configurations with two Li atoms on each side of the BP sheet, starting from (i) 2B-I and 2B-III structures with Li atoms on octagonal hole sites and either hexagonal hole sites or at bridges between hexagons and squares; (ii) 2B-II and 2B-IV structures, with Li atoms occupying only octagonal hole sites; and (iii) 2B-VII configuration with Li adsorbed only on hexagonal hole sites. The 2B-I configuration is stable at 300 K, and Li atoms vibrate near their equilibrium positions, with those located on octagonal hole sites showing larger vibration amplitudes. The 2B-III configuration turns into the first one, 2B-I, during the MD run, tending to align the Li atoms (half of them above and the other half below) by moving the Li atoms from the centers of the hexagons towards the bridges between hexagons and squares, as shown in Figure 10. On the other hand, the rest of the studied structures, namely, 2B-II, 2B-IV, and 2B-VII, remain stable during the 3 ps time spanned by the simulations. 

**Figure 11:**
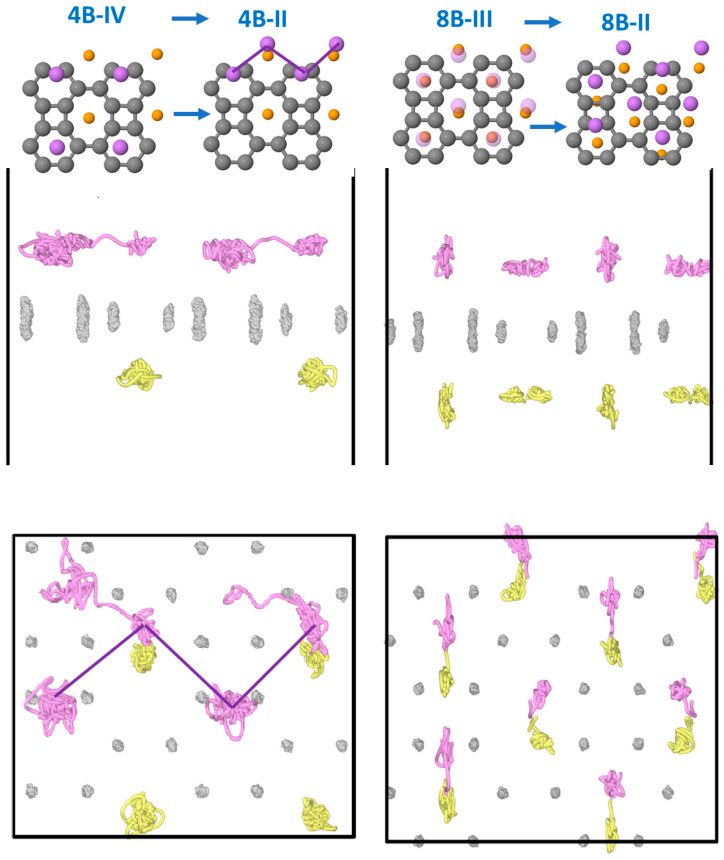
Projections of the atomic MD trajectories onto the xz plane (**upper graphs**) and the xy plane (**lower graphs**). Left side graphs: MD starting from structure 4B-IV; right side graphs: MD starting from structure 8B-III. Grey lines: C atoms. Pink lines: Li atoms above the BP sheet. Yellow lines: Li atoms below the BP sheet.

In the case of 8 Li atoms adsorbed on the BP sheet, 4 on each side, we performed two MD calculations. The 4B-I configuration, where two Li zigzag chains along the y direction are formed, is found to be stable during the MD run. On the contrary, the 4B-IV configuration, with Li atoms on hexagons on one side and octagons on the other, is not stable during the MD run; the lower part does not change, whereas, in the upper part, the Li atoms initially in the hexagonal hole sites move, through several phases, towards octagonal hole sites leading to structure 4B-II. Figure 11 shows the atomic trajectories, with pink lines denoting the atoms above the BP sheet and yellow lines denoting those placed below. It is observed that the C atoms mainly vibrate in the z-direction, and the Li atoms below the sheet in the octagonal positions vibrate around their equilibrium positions quite isotropically. The displacement of the Li atoms above the BP sheet that was initially on the hexagonal hole sites occurs in two phases: the one on the right-hand side moves towards the octagon first, and the one on the left migrates later. A movie in the Supplementary Information (movie-4B.mp4) illustrates the time evolution of the structure.

**Figure 12:**
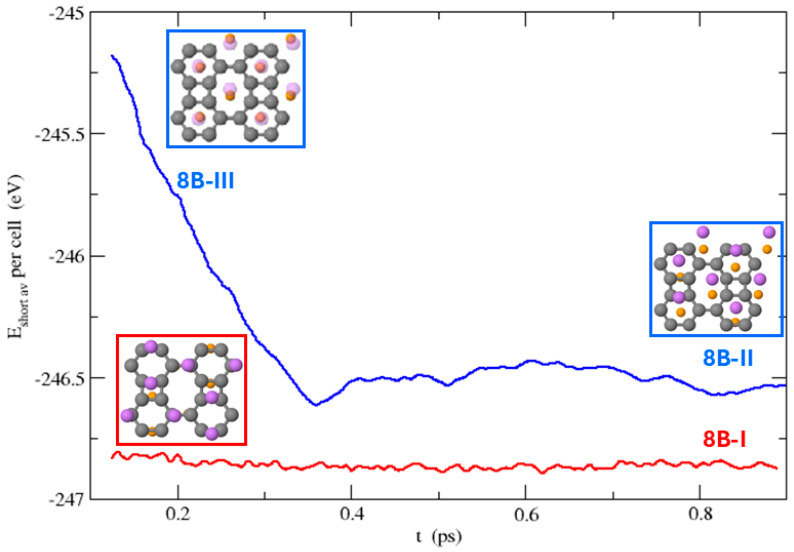
Time evolution of the short-time averages of the potential energy during the MD runs that start from the stable structure 8B-I (red line) and from the dynamically unstable structure 8B-III (red line) that evolves to 8B-II.

For *n* = 8, with sixteen Li atoms, the Li atoms only move slightly in the simulation starting from the configuration 8B-I. The structure 8B-III does not remain stable during the MD simulation, as some Li atoms that were originally on hexagonal hole sites move towards the hexagon-square bridges, and those above and below octagonal hole sites move antisymmetrically in the y-direction, ending in configuration 8B-II (see video movie-8B.mp4 in the Supplementary Information and Figure 12). 

## 4. Conclusions

We performed a comprehensive investigation of Li adsorption on the BP sheet using ab initio DFT calculations. A broad range of possible adsorption sites was explored, including mixed configurations such as hexagon-octagon and hexagon-square arrangements. Additionally, a quantum topological analysis of charge density and the electron localization function was performed to reveal the bonding patterns associated with different local environments induced by Li adsorption. To further evaluate the structural stability under practical conditions, ab initio MD simulations were carried out for selected configurations at room temperature.

Our results show that a single Li atom per unit cell preferentially binds to hexagonal, octagonal, and square hole sites in the BP supercell with close binding energies. When placing one Li atom on each side of the sheet, the most stable configuration involves Li atoms positioned in opposing hexagons. For two Li atoms on a single side, octagonal hole sites are preferred, whereas, for two Li atoms on each side, the most stable configuration features Li atoms aligned across the layer, with those on one side occupying bridge positions over square sites. At higher concentrations, the adsorption of four Li atoms on a single side leads to the formation of extended Li wires in a zigzag pattern, with Li atoms occupying bridge positions over octagonal and square hole sites. A similar wire-like arrangement emerges when four Li atoms are adsorbed on both sides of the BP sheet. With eight Li atoms, a quasi-monolayer structure forms, where Li atoms initially located on octagonal hole sites shift toward bridge positions while the other Li atoms remain on square hole sites. When eight Li atoms are adsorbed on each side of the sheet, a Li monolayer is formed on both surfaces. Our quantum-chemical topology analysis of the charge density and the electron localization function confirms the formation of these Li wires and monolayers, identifying metallic Li–Li bonding as a key stabilizing factor.

The most stable configurations identified in our study were not considered in previous works, which had predicted the absence of Li clustering. Our prediction about the emergence of Li wires and Li monolayers as Li concentration increases is a novel finding that should be taken into account in future research on potential applications of biphenylene-based materials. Furthermore, our calculated Li binding energies indicate that biphenylene-based anodes could offer Li storage capacities higher than those previously reported.

Ab initio MD simulations at room temperature confirmed the dynamic stability of these ground state structures, as thermal energy was insufficient to overcome the barriers between different configurations or induce structural transitions. Only in certain metastable configurations—when at least four Li atoms per unit cell were adsorbed—did structural rearrangements occur, leading to more stable configurations where Li monolayers persisted. This suggests that Li diffusion does not occur at temperatures up to 300 K, which may hinder the suitability of these materials as Li-ion battery anodes despite their high Li storage capacities.

In conclusion, our study highlights the necessity of a systematic and comprehensive approach that considers a broad range of possible configurations, along with an analysis of the dynamic stability at finite temperature, to fully assess the potential applications of biphenylene-based and possibly other C-based 2D materials.

## Figures and Tables

**Table 1:** Average electronic density (ρ), electronic energy (η), and Laplacian of the density ($\nabla^{2}\rho$) at the BCPs for the Li-C and Li-Li bonds encountered in the structure of BP decorated with 2 Li atoms (2A-I), 4 Li atoms (4A-V and 4A-I), and 8 Li atoms (8A-I). Numbers in parentheses correspond to the average height of the BCPs with respect to the average height of the BP layer. The results are in 10^−3^ a.u. for the BCPs and in Å for the relative height.

				Li-C						Li-Li		
	Square			Hexagon			Octagon					
	ρ	η	$\nabla^{2}\rho$	ρ	η	$\nabla^{2}\rho$	ρ	η	$\nabla^{2}\rho$	ρ	η	$\nabla^{2}\rho$
2A-I	--			--			19.7	4.7	111.0			
							(0.97)					
4A-V	--			19.7	3.7	98.0	16.8	4.7	85.0	--		
				(1.17)			(1.07)					
4A-I	21.0	5.0	135.0	--			19.5	5.5	135.0	8.0	−0.8	4.3
	(1.21)						(1.23)			(2.03)		
8A-I	19.4	4.0	107.5	--			21.3	5.3	121.0	7.2	−0.8	0.0
	(1.23)						(1.22)			(2.20)		

## Data Availability

The data that support the findings of this study are available from the authors upon reasonable request.

## Supplementary Materials

The following supporting information can be downloaded at: https://www.mdpi.com/article/10.3390/nano15090700/s1. Video V1: (Color online) Video illustrating the evolution from the 8A-II configuration, with Li atoms on hexagons and octagons, to the 8A-I configuration (movie-8A.mp4). Video V2: (Color online) Video illustrating the evolution from the 4B-IV configuration, with Li atoms on hexagons on one side and octagons on the other, to the 4B-II configuration (movie-4B.mp4). Video V3: (Color online) Video illustrating the evolution from the 8B-III configuration, with Li atoms on hexagons and octagons, to the 8B-II configuration (movie-8B.mp4).
