# Supplementary material for: Ab Initio Study of the Structures, Bonding Interactions, and Thermal Stability of the Li-Decorated 2D Biphenylene Sheet
Source: Nanomaterials (Basel). 2025 May 7;15(9):700. doi: 10.3390/nano15090700 (PMC12073348; doi:10.3390/nano15090700)
Supplement: Supplementary file 1 [file nanomaterials-15-00700-s001.zip › nanomaterials-3584197-supplementary-final.pdf]

nanomaterials

MDPI

Article

Ab Initio Study of the Structures, Bonding Interactions, and Thermal Stability of the Li-Decorated 2D Biphenylene Sheet

María Begoña Torres [1,*], Alexandre Lebon [2], Luis Enrique González [3], Luis Javier Gallego [4,*] and Andrés Vega [3]

[1] Departamento de Matemáticas y Computación, Escuela Politécnica Superior, Universidad de Burgos, ES-09006 Burgos, Spain

[2] Laboratoire de Chimie Electrochimie Moléculaire et Chimie Analytique, Université de Brest, UMR CNRS 6521, F-29285 Brest, France; alexandre.lebon@univ-brest.fr

[3] Departamento de Física Teórica, Atómica y Óptica, Facultad de Ciencias, Universidad de Valladolid, ES-47011 Valladolid, Spain; luisenrique.gonzalez@uva.es (L.E.G.); avega@uva.es (A.V.)

[4] Área de Física de la Materia Condensada, Departamento de Física de Partículas, Facultad de Física, Universidad de Santiago de Compostela, ES-15782 Santiago de Compostela, Spain

* Correspondence: begonia@ubu.es (M.B.T.); luisjavier.gallego@usc.es (L.J.G.)

Abstract: We performed an extensive study on the most stable structures, the electronic properties, and the thermal stability of the 2D biphenylene sheet decorated with Li atoms. Our structural results show that the Li storage capacity of biphenylene is much higher than that recently reported, which increases the interest in this 2D material as a promising anode material for Li-ion batteries, although Li diffusion is not expected at room temperature. Moreover, we found striking phenomena that had not been detected yet, such as the formation of Li zigzag wires and metallic Li monolayers on the biphenylene sheet beyond a certain coverage threshold. In our calculations, we use high-level density-functional theory, quantum chemical topology analysis, and ab initio molecular dynamics simulations. In particular, the latter methodology allows for confirming the stability of the predicted Li-decorated biphenylene structures at room-temperature conditions.

Keywords: 2D materials; biphenylene; Li-adsorption on biphenylene; structural properties; electronic properties; stability at room temperature; ab initio density-functional theory; ab initio molecular dynamics; quantum chemical topology; metallic bonding

Video V1: (Color online) Video illustrating the evolution from the 8A-II configuration, with Li atoms on hexagons and octagons, to the 8A-I configuration (movie-8A.mp4).

Video V2: (Color online) Video illustrating the evolution from the 4B-IV configuration, with Li atoms on hexagons on one side and octagons on the other, to the 4B-II configuration (movie-4B.mp4).

Video V3: (Color online) Video illustrating the evolution from the 8B-III configuration, with Li atoms on hexagons and octagons, to the 8B-II configuration (movie-8B.mp4).
